# Supplementary material for: Modeling glioblastoma heterogeneity as a dynamic network of cell states
Source: Mol Syst Biol. 2021 Sep 16;17(9):e10105. doi: 10.15252/msb.202010105 (PMC8444284; doi:10.15252/msb.202010105)
Supplement: Supplementary file 5 — Source Data for Figure 3 [file MSB-17-e10105-s001.zip › Figure3A_sourcedata/GSEA_3065/nosigdb_state3.GseaPreranked.1622718426028/RICHARDS_NOWAKOWSKI_SCIENCE_2017_IPC_NEN3_UPREG.html]

Details for gene set RICHARDS\_NOWAKOWSKI\_SCIENCE\_2017\_IPC\_NEN3\_UPREG[GSEA]

|  || Dataset | state3 |
| Phenotype | NoPhenotypeAvailable |
| Upregulated in class | na\_neg |
| GeneSet | RICHARDS\_NOWAKOWSKI\_SCIENCE\_2017\_IPC\_NEN3\_UPREG |
| Enrichment Score (ES) | -0.51520985 |
| Normalized Enrichment Score (NES) | -2.0589228 |
| Nominal p-value | 0.0 |
| FDR q-value | 1.2525253E-4 |
| FWER p-Value | 0.001 |
Table: GSEA Results Summary

  

Fig 1: Enrichment plot: RICHARDS\_NOWAKOWSKI\_SCIENCE\_2017\_IPC\_NEN3\_UPREG      
 Profile of the Running ES Score & Positions of GeneSet Members on the Rank Ordered List

  

| PROBE | GENE SYMBOL | GENE\_TITLE | RANK IN GENE LIST | RANK METRIC SCORE | RUNNING ES | CORE ENRICHMENT || 1 | SYNE2 |  |  | 16 | 0.529 | 0.0119 | No |
| 2 | CD63 |  |  | 26 | 0.480 | 0.0234 | No |
| 3 | PON2 |  |  | 63 | 0.389 | 0.0293 | No |
| 4 | RPN2 |  |  | 100 | 0.345 | 0.0341 | No |
| 5 | PTTG1IP |  |  | 137 | 0.315 | 0.0381 | No |
| 6 | CHD7 |  |  | 172 | 0.298 | 0.0419 | No |
| 7 | SRRM2 |  |  | 175 | 0.297 | 0.0494 | No |
| 8 | SEC11A |  |  | 205 | 0.281 | 0.0533 | No |
| 9 | MYO10 |  |  | 206 | 0.280 | 0.0607 | No |
| 10 | LAPTM4A |  |  | 208 | 0.278 | 0.0678 | No |
| 11 | PNN |  |  | 256 | 0.260 | 0.0691 | No |
| 12 | GON4L |  |  | 349 | 0.230 | 0.0643 | No |
| 13 | MSMO1 |  |  | 413 | 0.215 | 0.0625 | No |
| 14 | MAGI1 |  |  | 476 | 0.202 | 0.0605 | No |
| 15 | GJC1 |  |  | 516 | 0.196 | 0.0611 | No |
| 16 | LRP8 |  |  | 546 | 0.193 | 0.0627 | No |
| 17 | TMEM98 |  |  | 593 | 0.187 | 0.0622 | No |
| 18 | PGRMC2 |  |  | 618 | 0.184 | 0.0642 | No |
| 19 | ACLY |  |  | 624 | 0.183 | 0.0684 | No |
| 20 | GPC2 |  |  | 648 | 0.179 | 0.0704 | No |
| 21 | COL11A1 |  |  | 684 | 0.173 | 0.0708 | No |
| 22 | AASS |  |  | 696 | 0.170 | 0.0740 | No |
| 23 | GPRC5B |  |  | 730 | 0.166 | 0.0744 | No |
| 24 | SLC1A3 |  |  | 741 | 0.165 | 0.0776 | No |
| 25 | PAXBP1 |  |  | 745 | 0.165 | 0.0815 | No |
| 26 | CCT5 |  |  | 746 | 0.165 | 0.0858 | No |
| 27 | EZR |  |  | 759 | 0.162 | 0.0886 | No |
| 28 | MEGF10 |  |  | 762 | 0.162 | 0.0926 | No |
| 29 | EIF3A |  |  | 860 | 0.152 | 0.0852 | No |
| 30 | CACNA2D1 |  |  | 909 | 0.148 | 0.0834 | No |
| 31 | ADAR |  |  | 991 | 0.139 | 0.0776 | No |
| 32 | DLL3 |  |  | 999 | 0.138 | 0.0804 | No |
| 33 | MLC1 |  |  | 1033 | 0.136 | 0.0800 | No |
| 34 | TMX1 |  |  | 1053 | 0.134 | 0.0813 | No |
| 35 | FJX1 |  |  | 1071 | 0.133 | 0.0828 | No |
| 36 | MEX3A |  |  | 1101 | 0.130 | 0.0828 | No |
| 37 | CELSR2 |  |  | 1135 | 0.127 | 0.0822 | No |
| 38 | DAAM1 |  |  | 1211 | 0.121 | 0.0766 | No |
| 39 | ZNF292 |  |  | 1262 | 0.117 | 0.0737 | No |
| 40 | PPME1 |  |  | 1270 | 0.117 | 0.0760 | No |
| 41 | CCND2 |  |  | 1286 | 0.116 | 0.0772 | No |
| 42 | SLC4A7 |  |  | 1315 | 0.114 | 0.0769 | No |
| 43 | SRSF7 |  |  | 1328 | 0.113 | 0.0785 | No |
| 44 | FLNB |  |  | 1335 | 0.113 | 0.0807 | No |
| 45 | ZNF253 |  |  | 1364 | 0.111 | 0.0803 | No |
| 46 | LIMD1 |  |  | 1375 | 0.110 | 0.0820 | No |
| 47 | NONO |  |  | 1447 | 0.106 | 0.0764 | No |
| 48 | BAZ1A |  |  | 1603 | 0.097 | 0.0608 | No |
| 49 | ARID1A |  |  | 1676 | 0.093 | 0.0548 | No |
| 50 | C1orf61 |  |  | 1703 | 0.091 | 0.0541 | No |
| 51 | ZKSCAN1 |  |  | 1711 | 0.091 | 0.0557 | No |
| 52 | ZNF462 |  |  | 1733 | 0.090 | 0.0556 | No |
| 53 | CA12 |  |  | 1739 | 0.089 | 0.0573 | No |
| 54 | UBE2O |  |  | 1747 | 0.089 | 0.0588 | No |
| 55 | RIOK3 |  |  | 1799 | 0.087 | 0.0551 | No |
| 56 | SIPA1L2 |  |  | 1818 | 0.086 | 0.0552 | No |
| 57 | SDC2 |  |  | 1875 | 0.082 | 0.0508 | No |
| 58 | ACTR6 |  |  | 1948 | 0.078 | 0.0444 | No |
| 59 | POU2F1 |  |  | 1978 | 0.077 | 0.0430 | No |
| 60 | GOLGA4 |  |  | 2023 | 0.075 | 0.0398 | No |
| 61 | KAT6A |  |  | 2148 | 0.070 | 0.0271 | No |
| 62 | CTTN |  |  | 2280 | 0.065 | 0.0134 | No |
| 63 | RFTN2 |  |  | 2346 | 0.063 | 0.0075 | No |
| 64 | EIF3E |  |  | 2496 | 0.057 | -0.0085 | No |
| 65 | NPM1 |  |  | 2533 | 0.056 | -0.0113 | No |
| 66 | MED12L |  |  | 2601 | 0.053 | -0.0178 | No |
| 67 | QSER1 |  |  | 2621 | 0.053 | -0.0186 | No |
| 68 | IRGQ |  |  | 2642 | 0.052 | -0.0196 | No |
| 69 | UBAP2L |  |  | 2645 | 0.052 | -0.0185 | No |
| 70 | STAM |  |  | 2650 | 0.051 | -0.0176 | No |
| 71 | DPF2 |  |  | 2726 | 0.049 | -0.0251 | No |
| 72 | ZEB1 |  |  | 2764 | 0.048 | -0.0282 | No |
| 73 | BAZ2B |  |  | 2803 | 0.047 | -0.0314 | No |
| 74 | HEG1 |  |  | 2806 | 0.047 | -0.0304 | No |
| 75 | RBPJ |  |  | 2897 | 0.044 | -0.0398 | No |
| 76 | IST1 |  |  | 3002 | 0.041 | -0.0510 | No |
| 77 | LDHB |  |  | 3031 | 0.040 | -0.0532 | No |
| 78 | RRP8 |  |  | 3129 | 0.038 | -0.0636 | No |
| 79 | PAK3 |  |  | 3132 | 0.038 | -0.0628 | No |
| 80 | IVNS1ABP |  |  | 3239 | 0.035 | -0.0744 | No |
| 81 | JAKMIP2 |  |  | 3353 | 0.032 | -0.0868 | No |
| 82 | RGL1 |  |  | 3374 | 0.031 | -0.0883 | No |
| 83 | POU3F2 |  |  | 3388 | 0.031 | -0.0890 | No |
| 84 | AGO1 |  |  | 3413 | 0.030 | -0.0911 | No |
| 85 | ZFHX4 |  |  | 3437 | 0.029 | -0.0930 | No |
| 86 | SCRN1 |  |  | 3507 | 0.028 | -0.1003 | No |
| 87 | TFDP2 |  |  | 3647 | 0.024 | -0.1160 | No |
| 88 | SETBP1 |  |  | 3729 | 0.022 | -0.1249 | No |
| 89 | MDM2 |  |  | 3753 | 0.021 | -0.1271 | No |
| 90 | SALL1 |  |  | 3800 | 0.020 | -0.1319 | No |
| 91 | LZTS1 |  |  | 3801 | 0.020 | -0.1314 | No |
| 92 | ACIN1 |  |  | 4008 | 0.015 | -0.1552 | No |
| 93 | CSNK1A1 |  |  | 4049 | 0.014 | -0.1595 | No |
| 94 | BTG2 |  |  | 4070 | 0.013 | -0.1615 | No |
| 95 | SCHIP1 |  |  | 4109 | 0.012 | -0.1657 | No |
| 96 | EGR1 |  |  | 4134 | 0.012 | -0.1682 | No |
| 97 | VEZF1 |  |  | 4169 | 0.011 | -0.1719 | No |
| 98 | CBFA2T2 |  |  | 4198 | 0.010 | -0.1749 | No |
| 99 | SMAD5 |  |  | 4201 | 0.010 | -0.1749 | No |
| 100 | FOXN2 |  |  | 4204 | 0.009 | -0.1749 | No |
| 101 | GTF2I |  |  | 4264 | 0.008 | -0.1816 | No |
| 102 | NKAIN3 |  |  | 4346 | 0.005 | -0.1910 | No |
| 103 | TMTC2 |  |  | 4360 | 0.005 | -0.1923 | No |
| 104 | GDI2 |  |  | 4444 | 0.003 | -0.2020 | No |
| 105 | TCF3 |  |  | 4499 | 0.001 | -0.2083 | No |
| 106 | TMEM258 |  |  | 4509 | 0.001 | -0.2093 | No |
| 107 | PTPRS |  |  | 4549 | -0.000 | -0.2139 | No |
| 108 | NCOA5 |  |  | 4563 | -0.001 | -0.2154 | No |
| 109 | PSME4 |  |  | 4642 | -0.002 | -0.2245 | No |
| 110 | SYNRG |  |  | 4814 | -0.006 | -0.2444 | No |
| 111 | USP3 |  |  | 4815 | -0.006 | -0.2442 | No |
| 112 | ZNF232 |  |  | 4835 | -0.007 | -0.2463 | No |
| 113 | OXR1 |  |  | 4840 | -0.007 | -0.2466 | No |
| 114 | CELSR1 |  |  | 4969 | -0.010 | -0.2613 | No |
| 115 | DENND1B |  |  | 5119 | -0.014 | -0.2784 | No |
| 116 | CLASRP |  |  | 5123 | -0.014 | -0.2784 | No |
| 117 | EHBP1 |  |  | 5144 | -0.014 | -0.2804 | No |
| 118 | KIF26B |  |  | 5153 | -0.015 | -0.2809 | No |
| 119 | BCCIP |  |  | 5216 | -0.016 | -0.2878 | No |
| 120 | FGD4 |  |  | 5246 | -0.017 | -0.2907 | No |
| 121 | PSMA3 |  |  | 5498 | -0.024 | -0.3195 | No |
| 122 | FRMD4A |  |  | 5530 | -0.025 | -0.3225 | No |
| 123 | ZHX3 |  |  | 5639 | -0.028 | -0.3344 | No |
| 124 | FBL |  |  | 5681 | -0.029 | -0.3385 | No |
| 125 | ARMCX6 |  |  | 5693 | -0.030 | -0.3390 | No |
| 126 | TP53 |  |  | 5700 | -0.030 | -0.3389 | No |
| 127 | HDAC9 |  |  | 5716 | -0.030 | -0.3399 | No |
| 128 | PEX19 |  |  | 5768 | -0.032 | -0.3450 | No |
| 129 | MED27 |  |  | 5774 | -0.032 | -0.3448 | No |
| 130 | HES6 |  |  | 5805 | -0.033 | -0.3474 | No |
| 131 | PHB |  |  | 5852 | -0.034 | -0.3519 | No |
| 132 | MAP6 |  |  | 6061 | -0.042 | -0.3752 | No |
| 133 | IFT74 |  |  | 6066 | -0.042 | -0.3746 | No |
| 134 | SVIL |  |  | 6089 | -0.042 | -0.3761 | No |
| 135 | LHX2 |  |  | 6148 | -0.045 | -0.3817 | No |
| 136 | NOL4L |  |  | 6168 | -0.045 | -0.3828 | No |
| 137 | FANCF |  |  | 6171 | -0.045 | -0.3818 | No |
| 138 | MDK |  |  | 6242 | -0.048 | -0.3888 | No |
| 139 | CORO1C |  |  | 6385 | -0.052 | -0.4041 | No |
| 140 | CDC37L1 |  |  | 6398 | -0.052 | -0.4041 | No |
| 141 | PSMD5 |  |  | 6429 | -0.054 | -0.4062 | No |
| 142 | TFAP2C |  |  | 6436 | -0.054 | -0.4055 | No |
| 143 | METAP2 |  |  | 6486 | -0.056 | -0.4098 | No |
| 144 | DUSP12 |  |  | 6528 | -0.057 | -0.4131 | No |
| 145 | CHMP1B |  |  | 6554 | -0.059 | -0.4145 | No |
| 146 | FDFT1 |  |  | 6637 | -0.063 | -0.4225 | No |
| 147 | HNRNPC |  |  | 6643 | -0.063 | -0.4214 | No |
| 148 | CACUL1 |  |  | 6695 | -0.066 | -0.4257 | No |
| 149 | ETF1 |  |  | 6805 | -0.071 | -0.4366 | No |
| 150 | INSM1 |  |  | 6838 | -0.072 | -0.4385 | No |
| 151 | SEZ6L |  |  | 6920 | -0.077 | -0.4460 | No |
| 152 | DNM2 |  |  | 6942 | -0.078 | -0.4464 | No |
| 153 | GLUL |  |  | 6961 | -0.079 | -0.4465 | No |
| 154 | MED10 |  |  | 6991 | -0.080 | -0.4478 | No |
| 155 | NEDD4L |  |  | 7025 | -0.083 | -0.4495 | No |
| 156 | MMADHC |  |  | 7047 | -0.084 | -0.4498 | No |
| 157 | GPN1 |  |  | 7075 | -0.085 | -0.4507 | No |
| 158 | CCNB1IP1 |  |  | 7161 | -0.091 | -0.4583 | No |
| 159 | DNAJC8 |  |  | 7186 | -0.093 | -0.4587 | No |
| 160 | ZNF146 |  |  | 7204 | -0.094 | -0.4582 | No |
| 161 | NSMCE1 |  |  | 7206 | -0.094 | -0.4559 | No |
| 162 | TEAD2 |  |  | 7498 | -0.117 | -0.4870 | No |
| 163 | NAP1L1 |  |  | 7538 | -0.121 | -0.4884 | No |
| 164 | RGS16 |  |  | 7541 | -0.122 | -0.4855 | No |
| 165 | NFE2L2 |  |  | 7651 | -0.133 | -0.4948 | No |
| 166 | EEF1D |  |  | 7817 | -0.154 | -0.5101 | Yes |
| 167 | LINC00667 |  |  | 7832 | -0.156 | -0.5077 | Yes |
| 168 | GLO1 |  |  | 7844 | -0.157 | -0.5049 | Yes |
| 169 | CDK2AP1 |  |  | 7846 | -0.158 | -0.5009 | Yes |
| 170 | SCAND1 |  |  | 7912 | -0.169 | -0.5041 | Yes |
| 171 | ENC1 |  |  | 7921 | -0.170 | -0.5006 | Yes |
| 172 | ENY2 |  |  | 8028 | -0.189 | -0.5081 | Yes |
| 173 | ZC3H15 |  |  | 8090 | -0.202 | -0.5099 | Yes |
| 174 | CHMP2A |  |  | 8134 | -0.213 | -0.5094 | Yes |
| 175 | SKP1 |  |  | 8173 | -0.220 | -0.5081 | Yes |
| 176 | C8orf59 |  |  | 8184 | -0.223 | -0.5035 | Yes |
| 177 | NFIX |  |  | 8192 | -0.226 | -0.4984 | Yes |
| 178 | RPL3 |  |  | 8247 | -0.238 | -0.4985 | Yes |
| 179 | C19orf70 |  |  | 8289 | -0.255 | -0.4967 | Yes |
| 180 | GSTP1 |  |  | 8296 | -0.257 | -0.4907 | Yes |
| 181 | MAD2L2 |  |  | 8416 | -0.301 | -0.4968 | Yes |
| 182 | PKM |  |  | 8420 | -0.301 | -0.4893 | Yes |
| 183 | RPL18 |  |  | 8424 | -0.304 | -0.4817 | Yes |
| 184 | RPL10 |  |  | 8426 | -0.304 | -0.4739 | Yes |
| 185 | ACTB |  |  | 8444 | -0.312 | -0.4677 | Yes |
| 186 | RPL13A |  |  | 8472 | -0.325 | -0.4624 | Yes |
| 187 | RPS24 |  |  | 8521 | -0.347 | -0.4590 | Yes |
| 188 | RPS27L |  |  | 8528 | -0.351 | -0.4505 | Yes |
| 189 | RPL23 |  |  | 8539 | -0.357 | -0.4423 | Yes |
| 190 | C12orf57 |  |  | 8557 | -0.368 | -0.4347 | Yes |
| 191 | SUMO2 |  |  | 8573 | -0.383 | -0.4265 | Yes |
| 192 | PDCD5 |  |  | 8583 | -0.387 | -0.4175 | Yes |
| 193 | TMEM158 |  |  | 8586 | -0.391 | -0.4075 | Yes |
| 194 | RPL21 |  |  | 8588 | -0.393 | -0.3973 | Yes |
| 195 | RPL8 |  |  | 8602 | -0.405 | -0.3883 | Yes |
| 196 | RPS27A |  |  | 8620 | -0.422 | -0.3793 | Yes |
| 197 | BTF3 |  |  | 8627 | -0.432 | -0.3687 | Yes |
| 198 | RPS27 |  |  | 8638 | -0.441 | -0.3584 | Yes |
| 199 | HMGN2 |  |  | 8644 | -0.449 | -0.3472 | Yes |
| 200 | RPS16 |  |  | 8645 | -0.450 | -0.3355 | Yes |
| 201 | RPS19 |  |  | 8648 | -0.453 | -0.3239 | Yes |
| 202 | RPL4 |  |  | 8653 | -0.458 | -0.3124 | Yes |
| 203 | HIGD2A |  |  | 8654 | -0.458 | -0.3005 | Yes |
| 204 | RPS5 |  |  | 8656 | -0.460 | -0.2886 | Yes |
| 205 | RPS18 |  |  | 8658 | -0.465 | -0.2766 | Yes |
| 206 | RPS25 |  |  | 8661 | -0.467 | -0.2646 | Yes |
| 207 | POLR2L |  |  | 8664 | -0.468 | -0.2526 | Yes |
| 208 | RPL35A |  |  | 8679 | -0.492 | -0.2414 | Yes |
| 209 | COMMD6 |  |  | 8683 | -0.496 | -0.2289 | Yes |
| 210 | RPL13 |  |  | 8685 | -0.499 | -0.2160 | Yes |
| 211 | H3F3B |  |  | 8687 | -0.504 | -0.2029 | Yes |
| 212 | RPL36 |  |  | 8694 | -0.512 | -0.1903 | Yes |
| 213 | RPLP2 |  |  | 8697 | -0.515 | -0.1771 | Yes |
| 214 | FABP5 |  |  | 8699 | -0.518 | -0.1637 | Yes |
| 215 | RPLP0 |  |  | 8703 | -0.524 | -0.1503 | Yes |
| 216 | RPS6 |  |  | 8718 | -0.559 | -0.1374 | Yes |
| 217 | RPS15 |  |  | 8723 | -0.582 | -0.1227 | Yes |
| 218 | RPL28 |  |  | 8727 | -0.604 | -0.1072 | Yes |
| 219 | RPLP1 |  |  | 8728 | -0.606 | -0.0914 | Yes |
| 220 | RPS8 |  |  | 8730 | -0.621 | -0.0754 | Yes |
| 221 | RPSA |  |  | 8736 | -0.657 | -0.0588 | Yes |
| 222 | RPL12 |  |  | 8740 | -0.668 | -0.0417 | Yes |
| 223 | RPL41 |  |  | 8743 | -0.718 | -0.0232 | Yes |
| 224 | TMSB4X |  |  | 8748 | -0.920 | 0.0004 | Yes |
Table: GSEA details [plain text format]

  

Fig 2: RICHARDS\_NOWAKOWSKI\_SCIENCE\_2017\_IPC\_NEN3\_UPREG: Random ES distribution      
 Gene set null distribution of ES for **RICHARDS\_NOWAKOWSKI\_SCIENCE\_2017\_IPC\_NEN3\_UPREG**
